# The indoleamine 2,3-dioxygenase pathway controls complement-dependent enhancement of chemo-radiation therapy against murine glioblastoma

**DOI:** 10.1186/2051-1426-2-21

**Published:** 2014-07-07

**Authors:** Minghui Li, Aaron R Bolduc, Md Nasrul Hoda, Denise N Gamble, Sarah-Bianca Dolisca, Anna K Bolduc, Kelly Hoang, Claire Ashley, David McCall, Amyn M Rojiani, Bernard L Maria, Olivier Rixe, Tobey J MacDonald, Peter S Heeger, Andrew L Mellor, David H Munn, Theodore S Johnson

**Affiliations:** 1 GRU Cancer Center, Georgia Regents University, Augusta, Georgia, 30912, USA; 2 Program in Cancer immunology, Inflammation and Tolerance (CIT), Georgia Regents University, Augusta, GA, USA; 3 Medical College of Georgia Department of Pediatrics, Georgia Regents University, 1120 Fifteenth Street, Augusta, GA CN-4141A, USA; 4 Department of Surgery, Georgia Regents University, Augusta, GA, USA; 5 Department of Neurology, Georgia Regents University, Augusta, GA, USA; 6 Department of Pathology, Georgia Regents University, Augusta, GA, USA; 7 Department of Neurosurgery, Georgia Regents University, Augusta, GA, USA; 8 Department of Medicine, Georgia Regents University, Augusta, GA, USA; 9 College of Allied Health Sciences Department of Medical Laboratory, Imaging & Radiologic Sciences, Georgia Regents University, Augusta, GA 30912, USA; 10 Aflac Cancer & Blood Disorders Center, Children’s Healthcare of Atlanta, Emory University School of Medicine, Atlanta, GA 30322, USA; 11 Department of Medicine, Division of Nephrology, The Immunology Institute, New York, NY 10025, USA; 12 Recanati-Miller Transplant Institute, Icahn School of Medicine at Mount Sinai, New York, NY 10025, USA

**Keywords:** IDO, Indoleamine, Complement, Tumor, Immunotherapy, Chemotherapy, Radiation therapy, Indoximod, Glioblastoma, NLG919

## Abstract

**Background:**

Indoleamine 2,3-dioxygenase (IDO) is an enzyme with immune-suppressive properties that is commonly exploited by tumors to evade immune destruction. Anti-tumor T cell responses can be initiated in solid tumors, but are immediately suppressed by compensatory upregulation of immunological checkpoints, including IDO. In addition to these known effects on the adaptive immune system, we previously showed widespread, T cell-dependent complement deposition during allogeneic fetal rejection upon maternal treatment with IDO-blockade. We hypothesized that IDO protects glioblastoma from the full effects of chemo-radiation therapy by preventing vascular activation and complement-dependent tumor destruction.

**Methods:**

To test this hypothesis, we utilized a syngeneic orthotopic glioblastoma model in which GL261 glioblastoma tumor cells were stereotactically implanted into the right frontal lobes of syngeneic mice. These mice were treated with IDO-blocking drugs in combination with chemotherapy and radiation therapy.

**Results:**

Pharmacologic inhibition of IDO synergized with chemo-radiation therapy to prolong survival in mice bearing intracranial glioblastoma tumors. We now show that pharmacologic or genetic inhibition of IDO allowed chemo-radiation to trigger widespread complement deposition at sites of tumor growth. Chemotherapy treatment alone resulted in collections of perivascular leukocytes within tumors, but no complement deposition. Adding IDO-blockade led to upregulation of VCAM-1 on vascular endothelium within the tumor microenvironment, and further adding radiation in the presence of IDO-blockade led to widespread deposition of complement. Mice genetically deficient in complement component C3 lost all of the synergistic effects of IDO-blockade on chemo-radiation-induced survival.

**Conclusions:**

Together these findings identify a novel mechanistic link between IDO and complement, and implicate complement as a major downstream effector mechanism for the beneficial effect of IDO-blockade after chemo-radiation therapy. We speculate that this represents a fundamental pathway by which the tumor regulates intratumoral vascular activation and protects itself from immune-mediated tumor destruction.

## Background

Immune responses to established tumors, and particularly brain tumors, are commonly ineffective in controlling tumor progression [1,2]. Nonetheless, vaccines against glioblastoma-specific antigens have resulted in improved survival in clinical trials, and chemotherapy appears to enhance this beneficial effect [3,4]. Emerging evidence indicates that brain tumors are not irreversibly “immune-privileged,” but rather employ a number of regulatory mechanisms to prevent immune-mediated tumor destruction [2,5]. Identifying and therapeutically targeting these poorly understood immune regulatory mechanisms could enable induction of protective, endogenous anti-tumor immunity.

Indoleamine 2,3-dioxygenase (IDO), an enzyme with immunosuppressive properties, has been detected in a wide variety of human tumors, including glioblastoma, and in tumor draining lymph nodes [6-8]. High tumor expression of IDO is an independent risk factor for poor outcome in a variety of human cancers, including glioblastoma, and orthotopic syngeneic GL261 tumors grown in C57BL/6 mice were shown in 2012 to exhibit an IDO-rich tumor microenvironment that protects tumors from T cell attack [7].

Mechanistically, IDO has been shown to suppress T cell responses and to promote activation of regulatory T cells (Treg cells) in settings as diverse as mucosal tolerance, pregnancy, chronic infection and organ transplantation [reviewed in [9]]. In addition to these known effects on the adaptive immune system, we previously showed widespread, T cell-dependent complement deposition during allogeneic fetal rejection upon maternal treatment with IDO-blockade [10]. IDO-dependent generation of the bioactive tryptophan metabolite kynurenine is a potent vasodilator [11] that appears to be critically important in preventing tissue rejection in one allogeneic heart graft model [12,13]. Whether and how these mechanisms impact tumor growth are unclear, but the strong association between IDO expression and progressive tumor growth has led to ongoing early-phase trials to test whether targeting IDO has clinical efficacy.

In preclinical models, IDO-inhibitors were shown to have synergistic effects when administered with multiple classes of chemotherapy agents and radiation [14,15]. Early-phase clinical trials combining standard chemotherapy with IDO-inhibitor drugs, such as 1-methyl-D-tryptophan (D-1MT, indoximod) and NLG919, for treatment of refractory solid tumors are in progress. Such approaches have not been previously attempted for brain tumors, and specific mechanisms through which IDO regulates tumor progression under these conditions are not known. Based on previously documented effects of radiation on inflammation and vascular activation [1,16] and evidence that IDO-blockade has some effect on tumor therapy [14,15], we tested the hypothesis that the IDO protects glioblastoma from the full effects of chemo-radiation therapy by preventing vascular activation and complement-dependent tumor destruction.

## Results

### IDO-blockade synergizes with standard chemo-radiation therapy to enhance survival in mice with glioblastoma

Clinically, glioblastoma tumors are treated with surgical resection, followed by temozolomide (TMZ)-based chemotherapy plus local radiation. IDO-blockade has been shown to enhance the effects of both chemotherapy and radiation [14]. We tested the hypothesis that blocking IDO synergizes with TMZ plus radiation therapy (RT) to enhance survival in a murine model of glioblastoma. We employed the mouse glioblastoma cell line GL261 which becomes a highly aggressive tumor with short survival time, so treatment was focused on a single cycle of therapy, and a synergistic effect was defined as prolongation of survival relative to controls.

**Figure 1:**
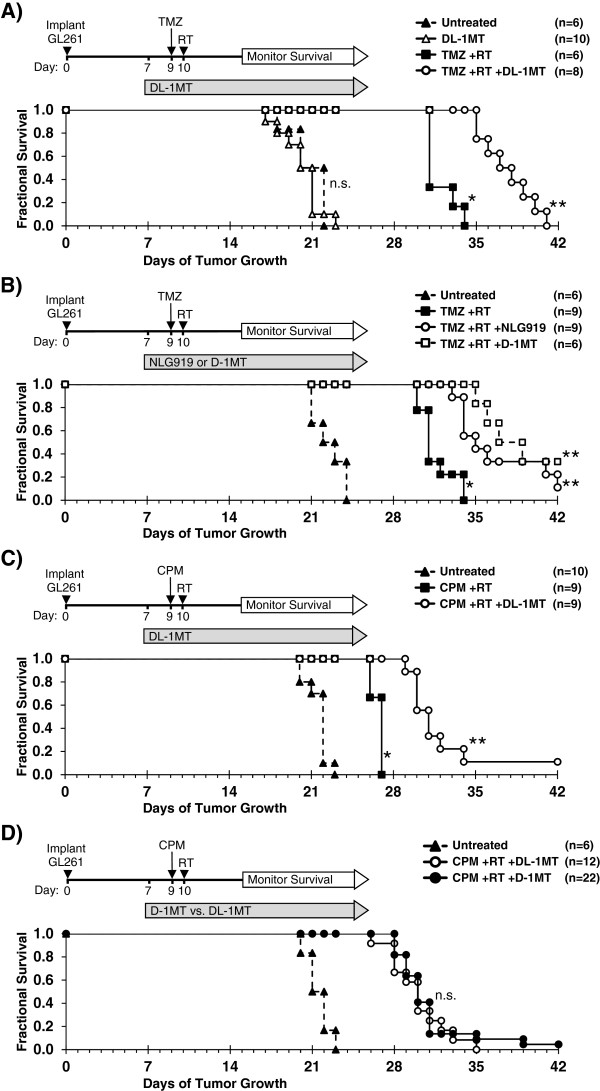
**IDO-blockade synergizes with chemo-radiation therapy.** Orthotopic GL261 glioblastoma tumors were implanted stereotactically into the right frontal lobes of syngeneic C57BL/6 host mice. Kaplan-Meier survival plots are shown, comparing mice treated with: **A**, temozolomide plus radiation (TMZ + RT) and with or without IDO-blockade using DL-1MT; **B**, TMZ + RT and with or without IDO-blockade using NLG919 or D-1MT; **C**, cyclophosphamide plus RT (CPM + RT) and with or without DL-1MT; or **D**, with CPM + RT plus either D-1MT or DL-1MT. IDO-blocking drugs (DL-1MT, 4 mg/mL; NLG919, 6 mg/mL; or D-1MT, 4 mg/mL) were supplied in drinking water continuously starting at day 7 after tumor implantation; chemotherapy (TMZ, 100 mg/kg, i.p.; or CPM, 100 mg/kg, i.p.) was given on day 9, and RT (500 cGy) was given on day 10. For reference, each survival plot contains a cohort of untreated mice, and mice treated with IDO-blockade using DL-1MT alone are shown in **A**. Cohort sizes (n) are indicated for each treatment group and represent pooled data from multiple experiments containing 1–3 mice from each group per experiment. n.s., not significant [vs. untreated mice **(A)**; or DL-1MT vs. D-1MT **(D)**]; *, *P* < 0.001 (vs. untreated mice); **, *P* < 0.002 (vs. mice treated with chemo-radiation alone), by log-rank test.

Seven days after injecting GL261 tumors, we treated recipients with or without the prototypical IDO-inhibitor compound, racemic 1-methyl-DL-tryptophan [17] (DL-1MT, 4 mg/mL in drinking water), followed by a single dose of TMZ (100 mg/kg) on day 9 and a single fraction of RT (500 cGy total-body irradiation) on day 10 (Figure 1A). Untreated mice reproducibly died after approximately 3 weeks of tumor growth (Additional file 1: Figure S1, range 18–24 days). Treatment with TMZ + RT alone improved overall survival relative to untreated mice (Figure 1A and B), adding 9 days to the median survival time (31 vs. 22 days). When we added DL-1MT to the TMZ + RT treatment, we observed a further prolongation of median survival by an additional 6.5 days (37.5 vs. 31 days) (Figure 1A). This is similar to the effect-size previously described as clinically significant in this glioblastoma model [18,19]. We also tested the effect of two IDO-inhibitor drugs that are currently in phase I clinical trials, 1-methyl-D-tryptophan (D-1MT) and NLG919. When combined with TMZ + RT, we observed that both NLG919 and D-1MT enhanced survival relative to mice treated with TMZ + RT alone (Figure 1B). The effect of these drugs was similar, as survival was not significantly different between groups treated with NLG919 plus TMZ + RT vs. D-1MT plus TMZ + RT.

Additional experiments in which we employed shielding to target the radiation dose to the cranium of the mice yielded results essentially identical to total-body irradiation (Additional file 1: Figure S2). In other control experiments, we observed that IDO-blockade had no survival-enhancing effect when combined with either TMZ alone (no RT; Additional file 1: Figure S3A), or RT alone (no chemotherapy; Additional file 1: Figure S3B). Together, these data indicate synergistic interactions among the three therapies when used together (IDO-blockade, TMZ, and RT), and the protective effect for the host was not influenced by the breadth of the radiation field.

### IDO-blockade synergizes with subtherapeutic cyclophosphamide plus radiation therapy to enhance survival

We also tested the ability of IDO-inhibitors to synergize with a cyclophosphamide (CPM)-based chemo-radiation regimen using the same glioblastoma model. CPM has been used to treat glioblastoma in the clinic [20] and has been widely studied in the context of immunotherapy [14,21]. We used a dose (100 mg/kg) that exhibited no therapeutic benefit as a single agent (Additional file 1: Figure S3C) and did not prolong survival when added to RT alone (Additional file 1: Figure S3B). However, when we added DL-1MT to CPM + RT we observed significantly prolonged survival compared to animals treated with CPM + RT alone (Figure 1C). Substituting the D-isomer of 1MT for the racemic mixture yielded the same results (Figure 1D). As with TMZ, IDO-blockade with CPM alone had no effect on survival (Additional file 1: Figure S3C). Thus, D-1MT and DL-1MT equivalently enhanced survival of mice with GL261 tumors treated with CPM + RT. In the subsequent experiments in this report, the D- and DL- preparations of 1MT were both used, and results were consistently equivalent.

### IDO target protein is expressed by GL261 tumors *in vivo*

GL261 tumors have been shown by others to express IDO protein *in vivo*, even in mice genetically deficient in the IDO-1 isoform [7,22]. We performed immunohistochemical staining on frozen sections of GL261 tumors to detect the presence of IDO protein in our system. We observed distinct patterns of IDO expression in tumor cells, in perivascular areas within and adjacent to tumors, and in peritumoral cells with astrocytic morphology (Additional file 1: Figure S4A). GL261 tumors were also found to express the IDO-2 isoform after 18 days of growth in either wild-type (WT) or IDO1-deficient host mice (Additional file 1: Figure S5). Because survival was improved when IDO-blockade was added to chemo-radiation therapy, we next analyzed GL261 tumors from treated host mice. We found that treatment of host mice with TMZ, TMZ + RT, or CPM + RT did not affect IDO target protein expression (Additional file 1: Figure S4B).

### Chemotherapy alone promotes perivascular accumulation of bone marrow-derived leukocyte cell populations within tumors

**Figure 2:**
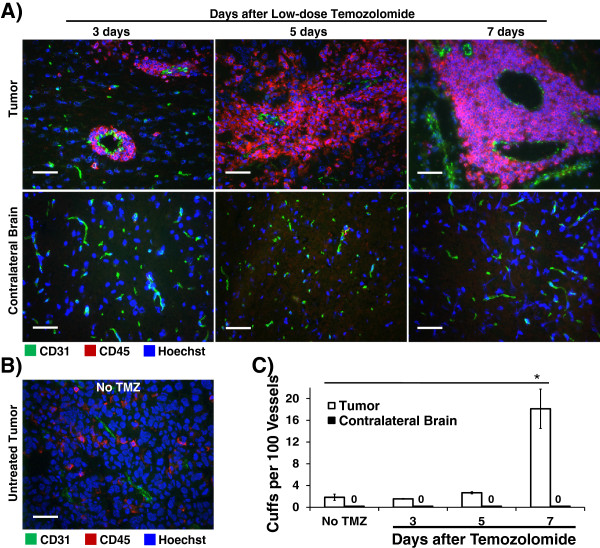
**Bone marrow-derived leukocytes aggregate around tumor blood vessels after chemotherapy treatment alone.** GL261 tumors from WT host mice were harvested 16 – 20 days after implantation, and: **A**, at 3, 5, or 7 days after a single low-dose temozolomide injection (25 mg/kg, i.p.) or **B**, from untreated mice. Tumors were frozen for immunohistochemical analysis of bone marrow-derived CD45-expressing leukocytes (red) and endothelial cells (CD31, green). Nuclei were counterstained with Hoechst (blue). Representative photomicrographs of tumors **(A and B)** and contralateral brain **(A, lower panels)** are shown. Original magnification, ×400; Scale bars, 25 μm. **C**, quantitative analysis was performed to determine the frequency of perivascular leukocyte cuffs per 100 vessels within tumors (white bars) and in contralateral brain (black bars) as a control. Photomicrographs were obtained in a grid pattern at magnification ×200 and analyzed for cuff frequency. Cuffs were counted if they were circumferential and at least 3 cells thick. For each mouse, at least 90 CD31-positive vessels from at least six 200X fields were analyzed for perivascular cuffs. Data are presented as mean ± SEM of 3 mice per time point, from 3 independent experiments. *, *P* < 0.0003, by ANOVA with Kruskal-Wallis test.

The above studies show that the protective effect of IDO-blockade requires both radiation and chemotherapy. To begin to dissect out the mechanisms through which each component of the therapeutic regimen contributed to the improved outcome, and to test the hypothesis that TMZ activates anti-tumor immunity and facilitates cellular infiltration into tumor parenchyma, we examined the brains from glioma-bearing mice treated with TMZ alone (Figure 2). For these studies, we chose a modest dose of TMZ (25 mg/kg) so that the peripheral leukocyte populations would not be reduced and the effects on tumor-infiltrating leukocytes would be visible. We performed immunohistochemical staining on frozen sections of brains harvested from glioma-bearing mice 3, 5, or 7 days after TMZ, using CD31 as a marker for vascular endothelium and CD45 as a marker for leukocytes derived from bone marrow.

Unexpectedly, instead of parenchymal infiltration of immune cells after chemotherapy treatment, we observed perivascular collections of CD45-positive leukocytes, as early as 3 days after chemotherapy treatment, which progressively increased over time (Figure 2A and C). In contrast, no perivascular leukocyte cuffs were observed in the contralateral (uninvolved) brain hemispheres. This vascular focus of leukocyte aggregation was due to chemotherapy, not the tumor itself, because only rare small collections were found in control, untreated, tumor-bearing mice (Figure 2B). Frequency analysis of these tissues for perivascular cuffs (defining a cuff as at least three layers of CD45-positive cells circumferentially around a CD31-positive vessel) demonstrated a significant increase in these large aggregates by day 7 after TMZ (Figure 2C). Finally, although TMZ can cause peripheral leukopenia, perivascular leukocyte cuffs were observed in tumors from mice treated with standard doses of either TMZ (100 mg/kg) or CPM (100 mg/kg) (Additional file 1: Figure S6).

The large perivascular cuffs from tumors harvested 7 days after TMZ (Additional file 1: Figure S7A) were predominantly comprised of CD68+ macrophages (CD45+) and microglia (CD45-lo), as well as substantial numbers of CD4+ T cells (Additional file 1: Figure S7B). A large proportion of the CD4 T cells co-stained for nuclear Foxp3, indicating a Treg cell phenotype (Additional file 1: Figure S7C). CD8 T cells were notably sparse in these leukocyte collections (Additional file 1: Figure S7B). Treating tumor-bearing mice with high dose chemotherapy plus RT caused the perivascular leukocyte cuffs and vessels to become disorganized with downregulation of endothelial CD31 expression (data not shown). Thus, while a single modest dose of TMZ alone had only minimal effect on survival, the intervention initiated a process of macrophage, microglia, and Treg cell aggregation at vascular sites within the tumor.

### Blocking IDO during chemotherapy enhances vascular activation

**Figure 3:**
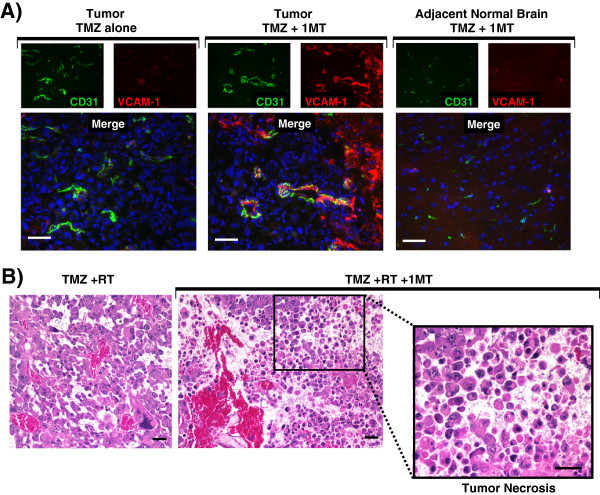
**IDO-blockade drives vascular activation after chemotherapy and tumor necrosis after chemo-radiation therapy. A**, GL261 tumors were grown in WT host mice treated with TMZ (100 mg/kg, i.p.) and with or without IDO-blockade using 1MT (4 mg/mL in drinking water). Tumors were harvested 5 days after chemotherapy (18 days after implantation) and frozen for immunohistochemical analysis of vascular cell adhesion molecule-1 (VCAM-1, red) on endothelial cells (CD31, green). Nuclei were counterstained with Hoechst (blue). Representative photomicrographs are shown of at least 3 mice per group, from at least 3 independent experiments. Original magnification, ×400; Scale bars, 25 μm. **B**, GL261 tumors were grown in WT host mice treated with TMZ (100 mg/kg, i.p.) + RT (500 cGy) and with or without 1MT (4 mg/mL in drinking water). Tumors were harvested in formalin 5 days after chemotherapy and stained with hematoxylin and eosin for assessment of tissue architecture. Call-out panel highlights an area of local tumor necrosis. Data are representative of at least 3 mice per group, from at least 3 independent experiments. Original magnification, ×200 (upper panels) and ×400 (lower call-out panel); Scale bars, 25 μm.

We next tested the hypothesis that adding IDO-blockade to temozolomide treatment activates tumor vessels (Figure 3). We treated glioma-bearing mice using TMZ, with or without 1MT. Tumors were harvested 5 days after treatment and immunohistochemistry was used to stain for vascular cell adhesion molecule-1 (VCAM-1) and CD31. Vessels within tumors of mice treated with TMZ alone expressed minimal VCAM-1. In contrast, when we added 1MT to TMZ treatment, we observed scattered but often intense VCAM-1 staining co-localized to CD31 indicative of endothelial cell upregulation (Figure 3A). In addition, we found that adjacent brain tissue was completely uninvolved (Figure 3A). Thus, while adding 1MT to TMZ treatment was completely ineffectual at enhancing survival in mice with glioblastoma (Additional file 1: Figure S3A), adding 1MT to TMZ selectively upregulated VCAM-1 on endothelial cells in vessels within the tumor, and this vascular activation was not found elsewhere in the brain.Because IDO-blockade had only provided synergistic prolongation of survival when it was combined with both chemotherapy and radiation, we next tested whether adding 1MT to TMZ + RT treatment altered the histological features of the glioma. Figure 3B shows hematoxylin and eosin (H&E) staining of formalin-fixed paraffin-embedded tumors harvested from WT mice treated with TMZ + RT, with or without 1MT. Control tumors treated with TMZ + RT alone were viable with erythrocytes confined to intact vascular structures (left panel). In contrast, tumors treated with IDO-blockade plus TMZ + RT had frequent areas of local tumor necrosis (Figure 3B). Similar effects were seen using cyclophosphamide in place of TMZ (data not shown).

### IDO regulates complement deposition within the tumor

**Figure 4:**
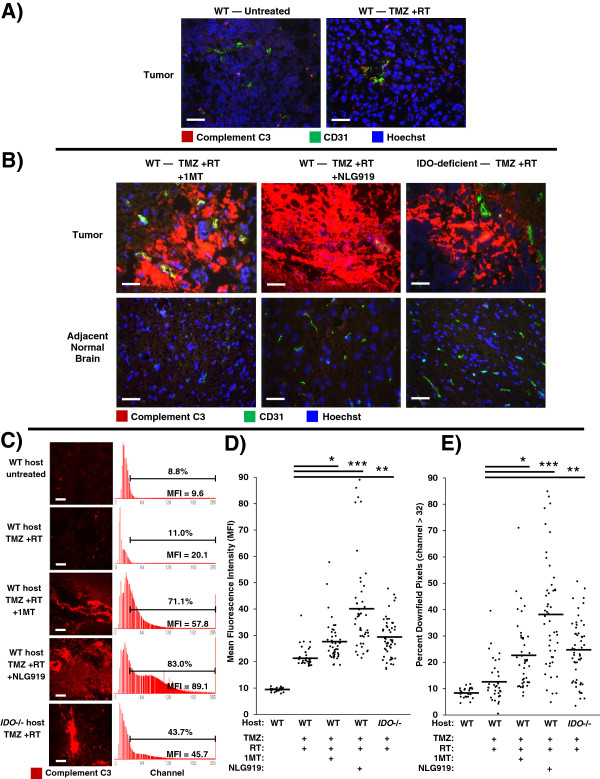
**Inhibition or absence of IDO triggers widespread complement deposition in tumors after chemo-radiation therapy.** GL261 tumors were grown in syngeneic host mice treated with or without IDO-blocking drugs (1MT, 4 mg/mL; or NLG919, 6 mg/mL) in drinking water starting on day 14, plus TMZ (100 mg/kg, i.p.) on day 16 and RT (500 cGy) on day 17. Tumors were harvested on day 18 and frozen for immunohistochemical analysis of complement component C3 deposition (red) endothelial cells (CD31, green), and nuclei (Hoechst, blue). **A**, representative photomicrographs of control tumors from WT host mice, either untreated or treated with TMZ + RT. **B**, representative photomicrographs of tumors and adjacent normal brain from WT host mice treated with either TMZ + RT + 1MT or TMZ + RT + NLG919; and from syngeneic IDO-deficient host mice treated with TMZ + RT. Data are representative of at least 3 mice per group, from at least 3 independent experiments. Original magnification, ×400; Scale bars, 25 μm. **C** to **E**, quantitative analysis of complement deposition. Photomicrographs of tumors stained for complement C3 (red) were obtained in a grid pattern at magnification ×400, and image analysis software was used to abstract fluorescence intensity histograms from each photomicrograph for quantitative analysis. **C**, Representative photomicrographs and histograms are shown. Scale bars, 25 μm. Histograms are labeled with mean fluorescence intensity (MFI) and the proportion of pixels occurring downfield from an arbitrarily-chosen negative threshold (channel thirty-two). Comparisons of MFI **(D)** and Percent Downfield Pixels **(E)** are shown with means represented by a solid bar. For each experimental group, at least 35 photomicrographs were analyzed from 3 separate mice pooled from 3 independent experiments. *, *P* < 0.02; **, *P* < 0.002; ***, *P* < 10^-6^ (vs. WT mice treated with TMZ + RT), by ANOVA with Kruskal-Wallis test.

We knew from prior work that IDO-blockade caused widespread complement deposition in the setting of allogeneic concepti being rejected after pregnant mice were treated with IDO-inhibitor drugs [10]. To test whether complement was an important mechanism in driving synergy between IDO-blockade and chemo-radiation therapy, we treated glioma-bearing WT mice with TMZ + RT, with or without either 1MT or NLG919. We assessed tumors using immunohistochemistry to co-stain for complement component C3 and CD31 (Figure 4).

We found that GL261 tumors from untreated mice did not stain for C3, and tumors from mice treated with TMZ + RT showed only scattered interstitial C3 staining (Figure 4A). However, when we combined TMZ + RT with IDO-blockade using either 1MT or NLG919, we observed extensive and confluent C3 deposition (Figure 4B). To test whether IDO was required for complement deposition, we treated IDO-deficient mice with TMZ + RT. We observed the same deposition of C3 in tumors from IDO-deficient host mice treated with TMZ + RT as we found in tumors from WT mice treated with IDO-blockade plus TMZ + RT (Figure 4B). We also found that C3 accumulation in tumors required the full combination of IDO-blockade with chemo-radiation therapy, as complement deposition was not seen in mice treated with IDO-blockade alone, IDO-blockade with TMZ (no RT), or IDO-blockade with RT (no TMZ) (Additional file 1: Figure S8). We also found that C3 deposition was strictly confined to the tumor microenvironment, and adjacent brain tissue was completely spared (Figure 4B). Thus, host IDO activity regulated complement deposition in glioblastoma tumors after chemo-radiation therapy, and complement deposition was highly selective and confined to the tumor itself.Image analysis software was used to quantitate photomicrographs of tumors stained for complement C3. Fluorescence-intensity histograms were generated for quantitative analysis of mean fluorescence intensity (MFI) and percent of downfield-gated (positive) pixels (Figure 4C). Tumors from mice with inactive IDO, either due to IDO-blockade or genetic IDO deficiency, treated with TMZ + RT had significantly increased complement C3 MFI (Figure 4D) and Percent Downfield Pixels (Figure 4E) compared to controls. Taken together, these data indicate that pharmacological blockade or genetic ablation of IDO allows TMZ + RT to trigger widespread C3 deposition in tumors consistent with local activation of the complement cascade, which is not observed when WT hosts are treated with TMZ + RT alone.

### Complement is mechanistically required for the beneficial effects of blocking IDO during chemo-radiation therapy

**Figure 5:**
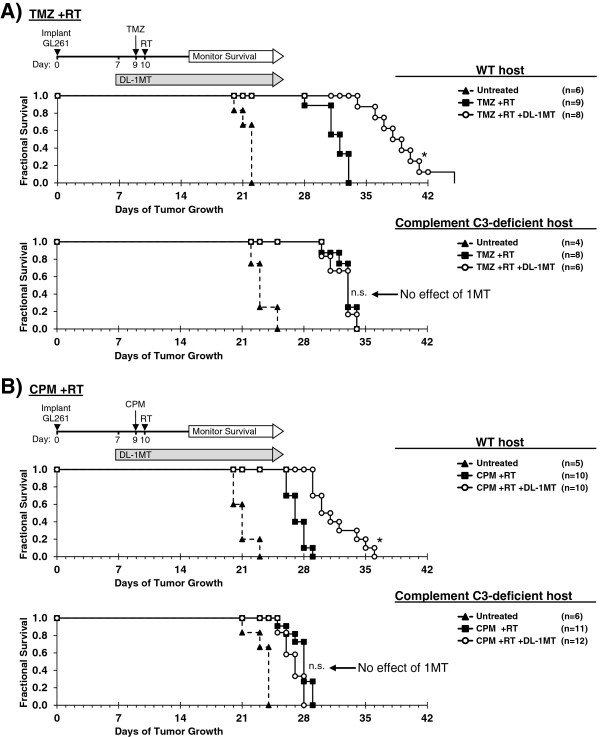
**Complement is mechanistically required for IDO-blockade to synergize with chemo-radiation therapy.** GL261 tumors were grown in syngeneic WT (upper panel) or complement C3-deficient (lower panel) host mice. Kaplan-Meier survival plots are shown, comparing mice treated with: **A**, temozolomide plus radiation (TMZ + RT) and with or without IDO-blockade using DL-1MT; or **B,** cyclophosphamide plus radiation (CPM + RT) and with or without DL-1MT. DL-1MT (4 mg/mL) was supplied in drinking water continuously starting at day 7 after tumor implantation; chemotherapy (TMZ, 100 mg/kg; or CPM, 100 mg/kg) was given on day 9, and RT (500 cGy) was given on day 10. For reference, each survival plot contains a cohort of untreated host mice. Cohort sizes (n) are indicated for each treatment group and represent pooled data from multiple experiments containing 1–3 mice from each group per experiment. *, *P* < 0.0001 (vs. mice treated with chemo-radiation alone); n.s., not significant (vs. mice treated with chemo-radiation alone), by log-rank test.

The preceding studies suggested that the extensive complement deposition observed in tumors when IDO was blocked represented the final effector stage of a multi-step inflammatory process. To test this hypothesis, we performed survival studies using tumor-bearing WT hosts or host mice lacking complement component C3 (*C3*-/-). We first verified that GL261 tumors grown in C3-deficient host mice retain the ability to express IDO at the protein level, using immunohistochemical staining (Additional file 1: Figure S9). For survival experiments, mice with GL261 tumors were treated with or without 1MT and chemo-radiation therapy, as shown in Figure 5. When we treated mice with chemo-radiation therapy alone (no IDO-inhibitor), we observed a comparable enhancement in survival relative to untreated controls, irrespective of whether the hosts were *C3*-/- or WT. Thus, C3 deficiency did not alter the underlying growth kinetics of the tumor, or the baseline response to chemo-radiation therapy.

As before, when we added 1MT to the treatment regimen, we observed the expected additional enhancement in survival for WT host mice. In contrast, we found that *C3*-/- host mice completely lost the beneficial survival effect of blocking IDO, and their survival curves became essentially identical with or without 1MT. Thus, the survival benefit provided by adding IDO-blockade to chemo-radiation therapy was strictly dependent on the ability of this combination to activate the complement system, and was abrogated in *C3*-/- host mice.

## Discussion

**Figure 6:**
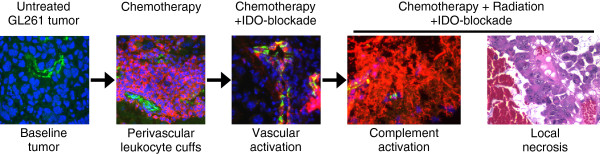
**Proposed sequence of events.** Recap of the proposed sequence of biological consequences of adding each component of therapy, culminating in local tumor necrosis, widespread complement deposition, and enhanced survival when RT is added to chemotherapy and IDO-blockade.

IDO protects tumors from anti-tumor immunity, although this has been thought to occur by suppression of T cell responses. We now show that blocking IDO results in C3-dependent tumor destruction, uncovering an unanticipated link between IDO and complement. In the absence of an IDO inhibitor (IDO activity intact), we did not observe complement deposition within tumors, regardless of whether RT was added to standard chemotherapy (Figure 4A and Additional file 1: Figure S8). Blocking IDO during chemo-radiation therapy led to widespread intratumoral deposition of C3 (Figure 4B), and the beneficial effect of IDO-blockade on survival was completely dependent on host C3 (Figure 5). Together, these data support the conclusion that inhibition of IDO unleashes local complement activation, which in turn affects tumor growth. The novel role for host IDO in controlling complement deposition in tumors and the key role that complement plays in damaging tumors when IDO is blocked have not been previously described.While the specific complement-dependent mechanisms require further study, we speculate that complement activation contributes to a vascular collapse in the tumor, with widespread “watershed” necrosis of tumor tissue that prolongs survival of the host animal. Any necrotic tumor cell death would occur in the context of IDO-blockade, and the resulting tumor debris may then activate adaptive immunity to mount a late-phase anti-tumor attack and develop immunological memory. We propose a model where each component of therapy (chemotherapy, IDO-blockade, radiation) contributes to a progressive sequence of biological consequences (Figure 6). We postulate that IDO normally imposes immunological quiescence on vessels in tumors, and even on the progressive perivascular leukocyte cuffs that form after chemotherapy. When IDO is blocked and tumors are further exposed to radiation, these may act together to sensitize the chemotherapy-induced perivascular cuff to allow innate inflammation, endothelial cell activation, widespread complement C3 deposition, and microangiopathic tumor destruction.

Tryptophan catabolism resulting in production of immuno-active kynurenine compounds and local tryptophan depletion can be mediated by either IDO or tryptophan 2,3-dioxygenase (TDO). We cannot rule out a contribution by TDO in these GL261 tumors, and there is no single-cell assay for production of tryptophan breakdown products. We have therefore been careful not to assume that inability to measure bioactive kynurenines equates to their absence in the relevant microenvironments during IDO-blocking treatment. However, there is a clear biological effect of blocking IDO with 1MT, which does not inhibit TDO [23], and of genetic IDO-1 deficiency (Figure 4B-E). In the short-term, IDO activity is important in suppressing complement deposition, and TDO does not appear to compensate for this when IDO is blocked during chemo-radiation therapy. Although TDO may be a problem for long-term host survival, which has clinical and therapeutic implications, the mechanistic question has been answered by IDO-deficient host mice which do develop complement deposition in tumors after chemo-radiation treatment. Nonetheless, it would be desirable to develop dual-specificity inhibitors for clinical application.

We used this model to study the short-term effects of adding IDO-blocking drugs to a single cycle of chemo-radiation therapy. The goal of any single cycle of chemo-radiation therapy is for the tumor to be smaller before the next cycle begins, and any human cancer patient would be treated with multiple cycles to compound the damage to the tumor. We speculate that, in our short-term model, adding IDO-blockade damages the tumor acutely via intensified inflammation and vasculitis, effectively amplifying the chemotherapy/radiation dose.

An emerging theme in cancer immunotherapy is the importance of vascular activation and targeting in mediating certain forms of tumor regression. Previous work in the B16 melanoma model has shown that despite evidence of peripheral anti-tumor T cell responses, tumor-specific vaccines are ineffective in causing tumor rejection, even when combined with CTLA-4-blockade and depletion of host Treg cells, until radiation is used to drive vascular activation [1]. Additional data from this model suggest that anti-tumor T cell responses are initiated in solid tumors, but immediately suppressed by compensatory upregulation of immunological checkpoints, including IDO [24]. Adoptive transfer of melanoma-specific CD8 T cells engineered to express IL-12 was found to cause tumor involution via collapse of tumor stroma and vasculature [25]. IL-12 is known to promote IFNγ secretion, which in turn can induce IDO expression in myeloid cell subsets [reviewed in [5]]. Preliminary experiments in our model show that the adaptive immune system is necessary for the formation of perivascular leukocyte collections after chemotherapy (unpublished data). It is also known from previous work that T cell interactions with antigen-presenting cells and/or endothelial cells initiate alternative pathway-dependent complement activation yielding local production of C3a and C5a [26-28]. These anaphylatoxins signal through their receptors on T cells to promote effector T cell activation and expansion [26,28] and inhibit the generation, function, and stability of Treg cells [29]. We propose that IDO-blockade during inflammatory chemo-radiation therapy drives a positive feedback loop between complement activation/deposition and pre-primed tumor-specific effector T cells, which activates and amplifies both processes.

## Conclusions

Based on the prominent connection between IDO and complement in settings as diverse as pregnancy and brain tumors, we propose that locally inhibiting the pro-inflammatory complement pathway is a fundamental mechanism by which IDO helps create immune tolerance. From a translational perspective, our data suggest that there is substantially more anti-tumor efficacy available from conventional chemo-radiation treatments if they are given in a setting in which IDO is blocked, thus allowing them to trigger the beneficial contribution of the complement pathway. Additional studies will be required to determine which components of the complement cascade are key mediators of the downstream biological effects of blocking IDO, which complement regulatory pathways are induced or maintained by IDO expression, and whether IDO activity directly affects complement production or activation.

## Methods

### Mice, glioma cell line, and reagents

C57BL/6, B6.129-Ido1tm1Alm/J (IDO1-deficient), and B6;129S4-C3^tm1Crr^/J (complement C3-deficient) mice were purchased from the Jackson Laboratory. Animal studies were approved by the Institutional Animal Care and Use Committee of Georgia Regents University. GL-261 cells were purchased from the National Institutes of Health Tumor Repository (Frederick, MD) and cultured in RPMI-1640 media supplemented with 10% fetal bovine serum, 4 mM L-glutamine, 100 IU/mL penicillin, and 100 μg/mL streptomycin (Corning Cellgro). IDO-inhibitor drugs 1-methyl-D-tryptophan (D-1MT, catalog no. 452483) and 1-methyl-L-tryptophan (L-1MT, catalog no. 447439) were purchased from Sigma-Aldrich, and NLG919 was a generous gift from Mario Mautino (NewLink Genetics). Primary antibodies for immunohistochemical studies were purchased from: Abcam [rabbit anti-mouse CD31 polyclonal antibody (catalog no. ab 28364)], eBioscience [rat anti-mouse CD45 (clone 30-F11)], AbD Serotec [rat anti-mouse VCAM-1 (clone MVCAM A(429))], and Cedarlane [rat anti-mouse complement C3 (clone RmC11H9, specific for native mouse C3 and breakdown products C3b, iC3b, and C3dg)]. Secondary immunohistochemistry reagents labeled with Alexa Fluor 488 or Cy3 were purchased from Jackson ImmunoResearch Laboratories, Inc.

### Tumors and animal treatments

Mice were anesthetized with 4% isoflurane, and the surgical plane of anesthesia was maintained with 2% isoflurane in oxygen. Mice were immobilized in a stereotactic frame (Stoelting Company) for tumor implantation. Briefly, the skull was shaved and exposed with a 0.5 cm skin incision. With antiseptic technique, 10^5^ GL261 cells (suspended in 3 μL RPMI-1640) were injected at the following coordinates with respect to the bregma on the right side (antero-posterior, -2 mm; medio-lateral, 2 mm; dorso-ventral, 3 mm). This placement reproducibly yielded tumor growth in a paracortical area of the posterolateral right frontal lobe. Tumor-bearing mice were treated with combinations of oral DL-1MT (2 mg/mL D-1MT mixed with 2 mg/mL L-1MT) in drinking water, D-1MT (4 mg/mL) in drinking water, NLG919 (6 mg/mL) in drinking water, intraperitoneal cyclophosphamide (Baxter), intraperitoneal temozolomide (Merck), and/or total-body radiation (500 cGy from a ^137^Cs source), as detailed in figure legends. Mice were observed daily, and sacrificed when they became ill or moribund, which defined the ethical surrogate endpoint as approved by our animal-use committee.

### Immunofluorescence staining

Tumor tissue was harvested, snap-frozen, and stored at -80°C until 5 μm sections were cut for immunohistochemical staining. Tissue sections were fixed with ethanol and acetone (1:1). Nonspecific binding to tissue was then blocked with normal mouse serum and serum matched to the secondary reagent host species. Sections were incubated with primary antibody followed by fluorescence-labeled secondary reagents. Nuclear counterstain was performed using Hoechst (catalog no. 14530, Sigma-Aldrich). Fluorescent microscopy (Olympus AX70 upright compound microscope) with a SPOT digital camera and software (SPOT Imaging Solutions) was used to generate photomicrographs.

### Quantitation of complement C3 staining

Photomicrographs of tumors stained for complement C3 (red) were obtained in a grid pattern at magnification ×400. Abobe Photoshop CS6 (Adobe Systems, Inc.) image analysis software was used to transform photomicrographs of complement C3-labeled tumors into fluorescence-intensity histograms for quantitative analysis of MFI and percent of downfield-gated (positive) pixels, defined as occurring above an arbitrarily-chosen negative threshold (above channel thirty-two). Histograms for reproduction in Figure 4 were generated by FastStone Image Viewer (FastStone Soft, 2013).

### Statistics

Statistical analysis was performed using NCSS 2007 statistical software (NCSS, LLC.). For survival data, Kaplan-Meier curves were analyzed by the log-rank test. For frequency analysis of perivascular cuff and quantitative analysis of complement C3 (MFI and Percent Downfield Pixels), ANOVA with Kruskal-Wallis test was used. Significance was defined as a *P* value less than 0.05. Time to ethical surrogate endpoint was treated as survival time for statistical analysis.

## Abbreviations

cGy: Centigray; CPM: Cyclophosphamide; D-1MT: 1-methyl-D-tryptophan; DL-1MT: Racemic 1-methyl-DL-tryptophan; IDO: Indoleamine 2,3-dioxygenase; MFI: Mean fluorescence intensity; RT: Radiation therapy; TDO: Tryptophan 2,3-dioxygenase; TMZ: Temozolomide; Treg cell: Regulatory T cell; VCAM-1: Vascular cell adhesion molecule-1; WT: Wild-type.

## Competing interests

ALM and DHM have intellectual property interests in the therapeutic use of IDO and IDO inhibitors, and receive consulting income and research support from NewLink Genetics, Inc. The other authors declare that they have no competing interests.

## Authors’ contributions

Conception and design: ML, DHM, TSJ. Development of methodology: ML, ARB, MNH, DNG, SD, AKB, KH, CA, AMR, BLM, DHM, TSJ. Acquisition of data: ML, ARB, DNG, SD, AKB, KH, CA, DM, TSJ. Analysis and interpretation of data: ML, AMR, BLM, OR, TJM, PSH, ALM, DHM, TSJ. Writing, review and/or revision of the manuscript: ML, ARB, MNH, DNG, SD, AKB, KH, CA, DM, AMR, BLM, OR, TJM, PSH, ALM, DHM, TSJ. Study supervision: TSJ. All authors read and approved the final manuscript.

## Supplementary Material

Additional file 1: Figure S1 Survival time is highly reproducible in untreated mice with intracranial GL261 tumors. **Figure S2.** Corporal shielding during radiation therapy does not affect synergy between IDO-blockade and chemo-radiation therapy. **Figure S3.** Neither chemotherapy alone nor radiation therapy alone are sufficient to drive synergy with IDO-blockade. **Figure S4.** IDO is expressed by GL261 tumors *in vivo*. **Figure S5.** IDO-2 is expressed by GL261 tumors *in vivo*. **Figure S6.** Perivascular leukocyte collections form around tumor blood vessels after treatment with standard-dose chemotherapy. **Figure S7.** Macrophages, microglia and regulatory CD4 T cells predominate in perivascular leukocyte aggregates after chemotherapy. **Figure S8.** Complement deposition does not occur without the combination of IDO-pathway blockade, chemotherapy and radiotherapy. **Figure S9.** IDO is expressed by GL261 tumors grown in complement C3-deficient host mice. **Methods for supplemental figures.** Click here for file
