# Supplementary material for: The indoleamine 2,3-dioxygenase pathway controls complement-dependent enhancement of chemo-radiation therapy against murine glioblastoma
Source: J Immunother Cancer. 2014 Jul 7;2:21. doi: 10.1186/2051-1426-2-21 (PMC4105871; doi:10.1186/2051-1426-2-21)
Supplement: Additional file 1: Figure S1 — Survival time is highly reproducible in untreated mice with intracranial GL261 tumors. Figure S2. Corporal shielding during radiation therapy does not affect synergy between IDO-blockade and chemo-radiation therapy. Figure S3. Neither chemotherapy alone nor radiation therapy alone are sufficient to drive synergy with IDO-blockade. Figure S4. IDO is expressed by GL261 tumors in vivo. Figure S5. IDO-2 is expressed by GL261 tumors in vivo. Figure S6. Perivascular leukocyte collections form around tumor blood vessels after treatment with standard-dose chemotherapy. Figure S7. Macrophages, microglia and regulatory CD4 T cells predominate in perivascular leukocyte aggregates after chemotherapy. Figure S8. Complement deposition does not occur without the combination of IDO-pathway blockade, chemotherapy and radiotherapy. Figure S9. IDO is expressed by GL261 tumors grown in complement C3-deficient host mice. Methods for supplemental figures. [file 2051-1426-2-21-S1.pdf]

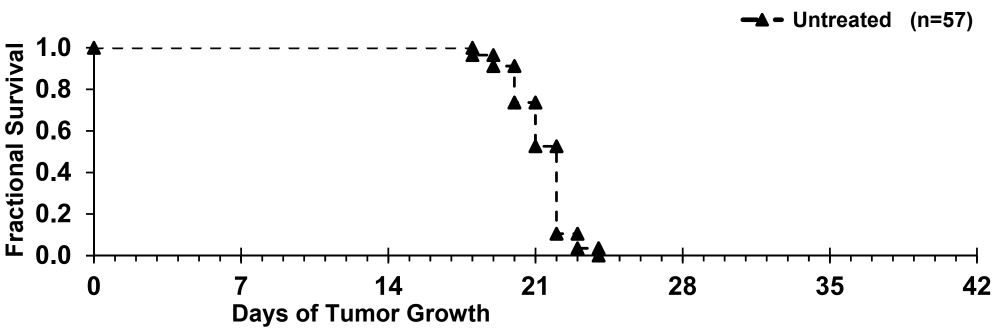

Figure S1. Survival time is highly reproducible in untreated mice with intracranial GL261 tumors. GL261 tumors were grown in syngeneic C57BL/6 host mice. The Kaplan-Meier fractional-survival plot shows robust reproducibility in survival time for 57 untreated animals pooled from more than 30 separate experiments.

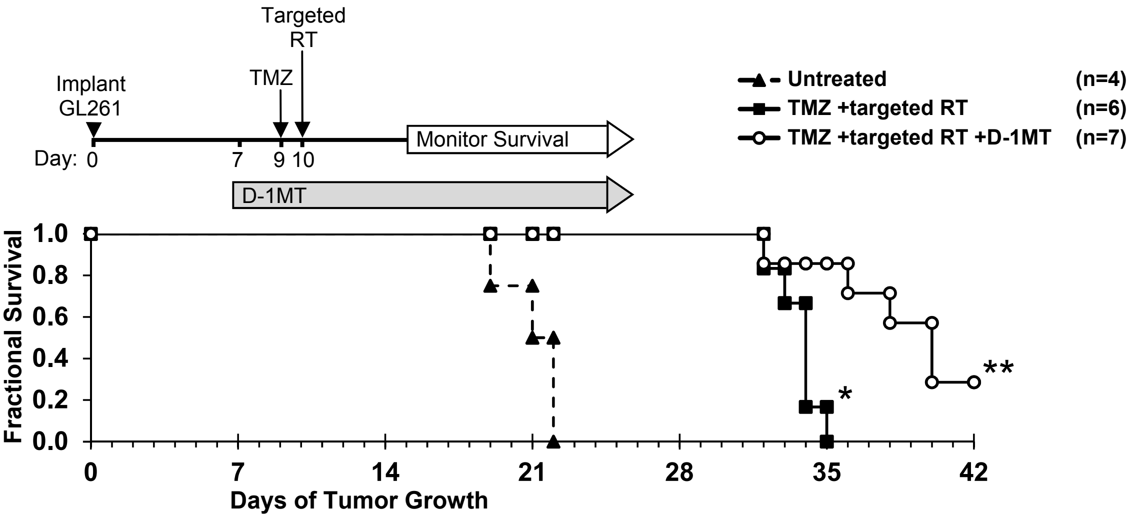

Figure S2. Corporal shielding during radiation therapy does not affect synergy between IDO-blockade and chemo-radiation therapy. GL261 tumors were grown in syngeneic C57BL/6 host mice. Kaplan-Meier survival plots are shown, comparing mice treated with temozolomide (TMZ) plus targeted cranial radiation (targeted RT) and with or without IDO-blockade using D-1MT. D-1MT (4 mg/mL) was supplied in drinking water continuously starting at day 7 after tumor implantation; TMZ (100 mg/kg, i.p.) was given on day 9, and targeted RT (500 cGy using corporal shielding) was given on day 10. A cohort of untreated mice is included for reference. Cohort sizes (n) are indicated for each treatment group and represent pooled data from multiple experiments containing 1-3 mice from each group per experiment. *, $P < 0.002$ (vs. untreated mice); **, $P < 0.006$ (vs. mice treated with chemo-radiation alone), by log-rank test.

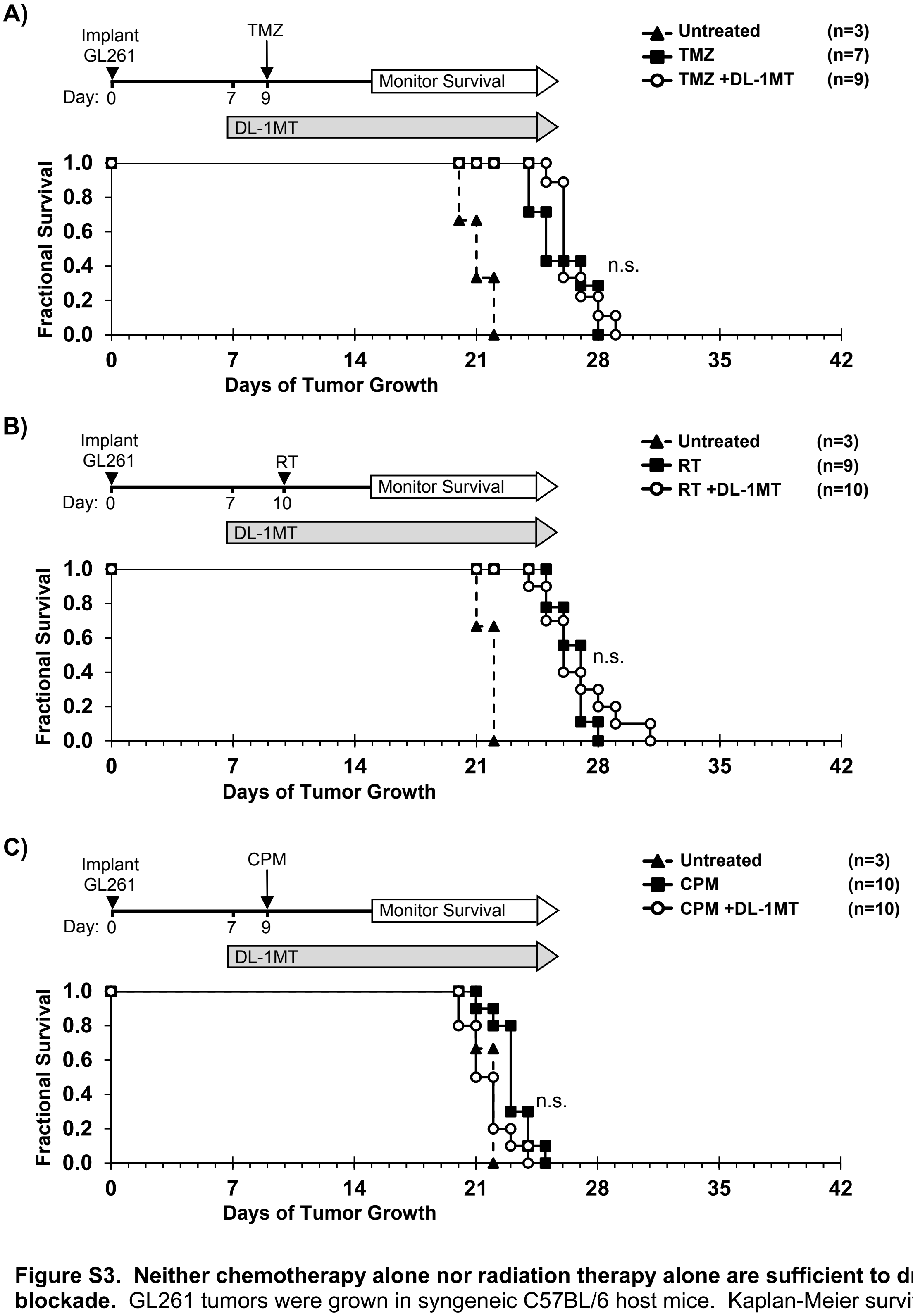

Figure S3. Neither chemotherapy alone nor radiation therapy alone are sufficient to drive synergy with IDO-blockade. GL261 tumors were grown in syngeneic C57BL/6 host mice. Kaplan-Meier survival plots are shown, comparing mice treated with or without DL-1MT plus either **A**, temozolomide (TMZ); **B**, radiation (RT); or **C**, cyclophosphamide (CPM). DL-1MT (4 mg/mL) was supplied in drinking water continuously starting at day 7 after tumor implantation; chemotherapy (**A**, TMZ, 100 mg/kg, i.p.; or **C**, CPM, 100 mg/kg, i.p.) was given on day 9, or **B**, RT (500 cGy) was given on day 10. For reference, each survival plot contains a cohort of untreated mice. Cohort sizes (n) are indicated for each treatment group and represent pooled data from multiple experiments containing 1-3 mice from each group per experiment. n.s., not significant (vs. mice treated with chemotherapy alone or radiation alone, respectively), by log-rank test.

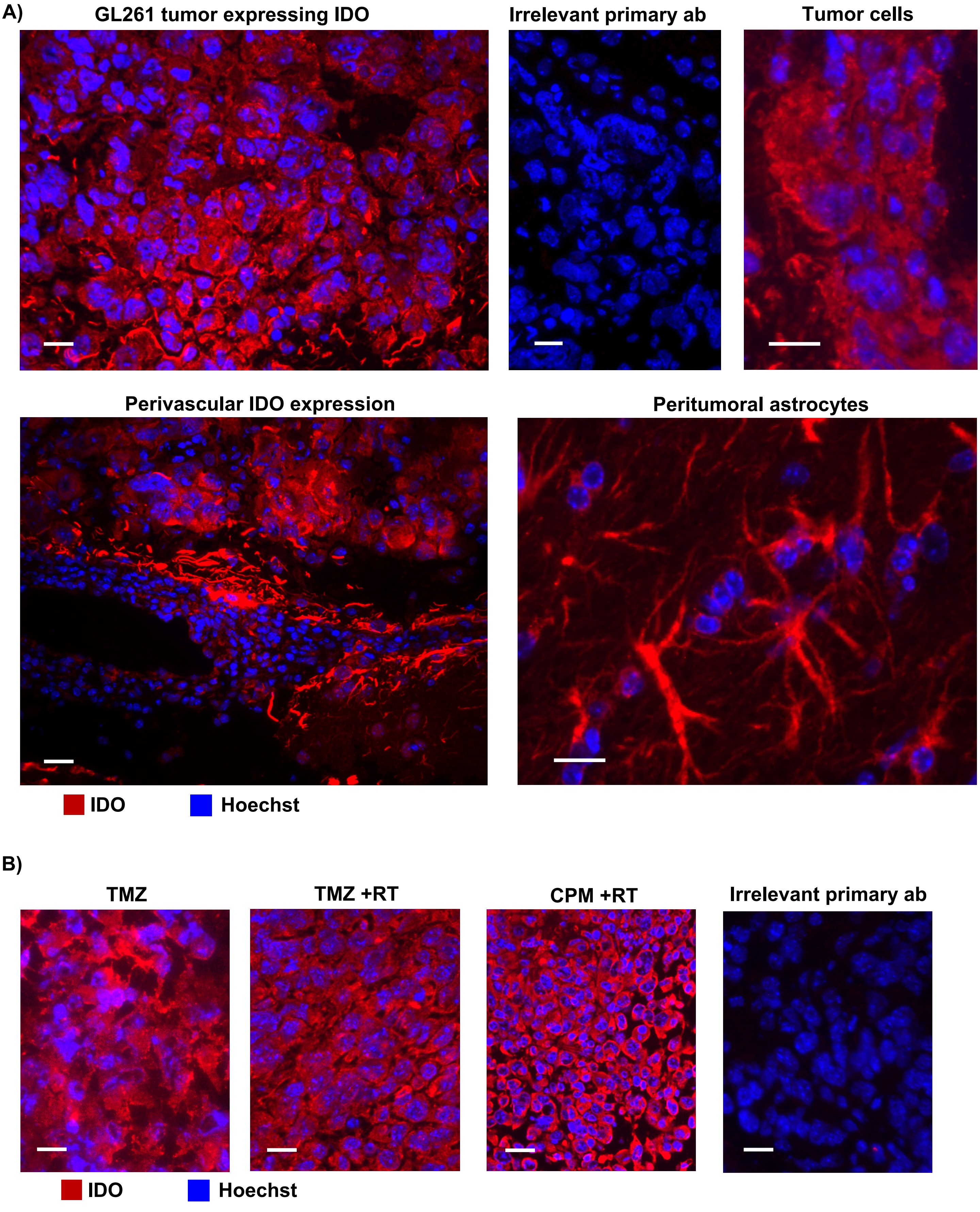

Figure S4. IDO is expressed by GL261 tumors *in vivo*.

Figure S4. IDO is expressed by GL261 tumors *in vivo*. GL261 tumors were grown in syngeneic C57BL/6 mice. **A**, tumors were harvested from untreated mice and frozen for immunohistochemical analysis using polyclonal rabbit anti-mouse IDO (red) and nuclear counterstain (Hoechst, blue). Examples of IDO-stained tumors cells (upper-right), perivascular areas (lower-left), and peritumoral astrocytic cells (lower-right) are shown. **B**, GL261 tumors were grown in syngeneic C57BL/6 host mice treated with temozolomide (TMZ, 100 mg/kg, i.p.), TMZ plus radiation (RT, 500 cGy), or cyclophosphamide (CPM, 100 mg/kg, i.p.) plus RT. Tumors were harvested 2 days after chemotherapy (for TMZ and TMZ+RT groups) or 5 days after chemotherapy (for CPM+RT group), and frozen for immunohistochemical staining with polyclonal rabbit anti-mouse IDO (red) and nuclear counterstain (Hoechst, blue). Results are representative of at least 3 mice in each group from multiple experiments. Tumors stained with irrelevant primary antibody are included for reference. Original magnification x400; Scale bars, 25 µm.

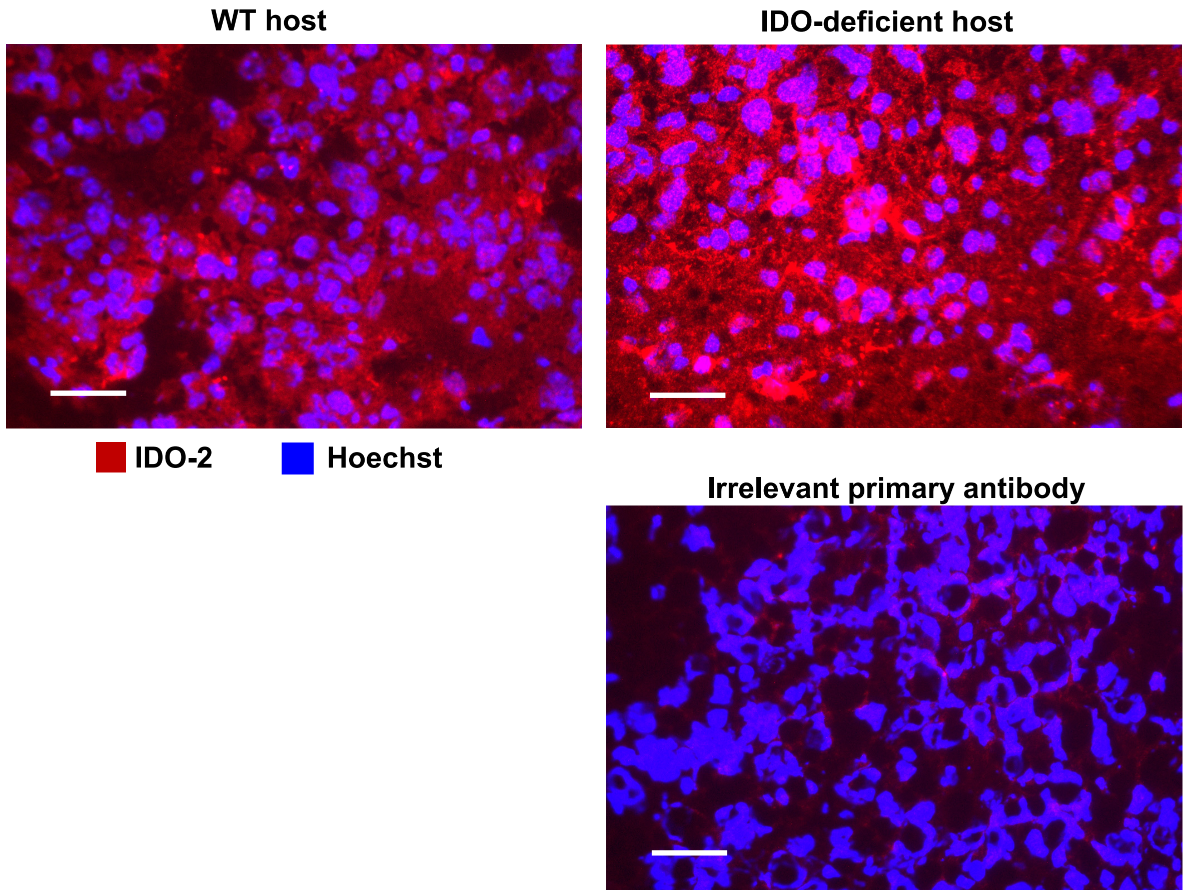

Figure S5. IDO-2 is expressed by GL261 tumors *in vivo*. GL261 tumors were harvested from untreated syngeneic C57BL/6 or IDO-deficient host mice and frozen for immunohistochemical staining with polyclonal rabbit anti-mouse IDO-2 (red) and nuclear counterstain (Hoechst, blue). Tumor stained with irrelevant primary antibody is included for reference. Results are representative of 3 mice in each group from 2 separate experiments. Original magnification x400; Scale bars, 50 μm.

A)

GL261 tumors from mice treated with temozolomide (100 mg/kg)

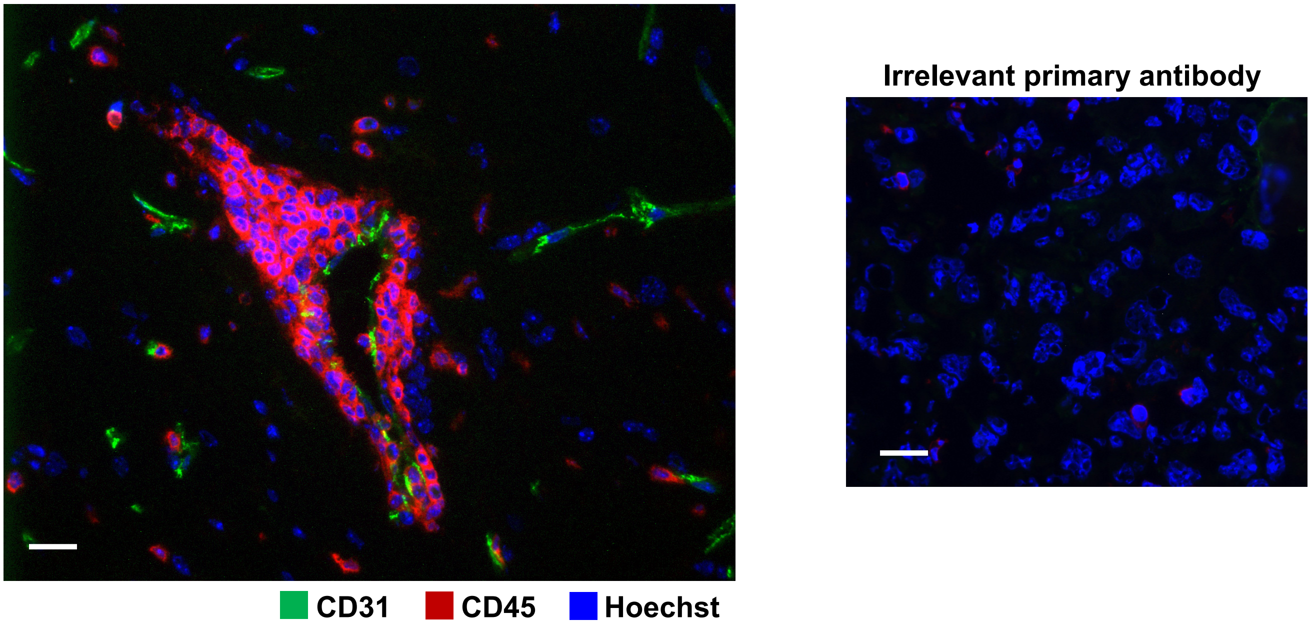

B)

GL261 tumors from mice treated with cyclophosphamide (100 mg/kg)

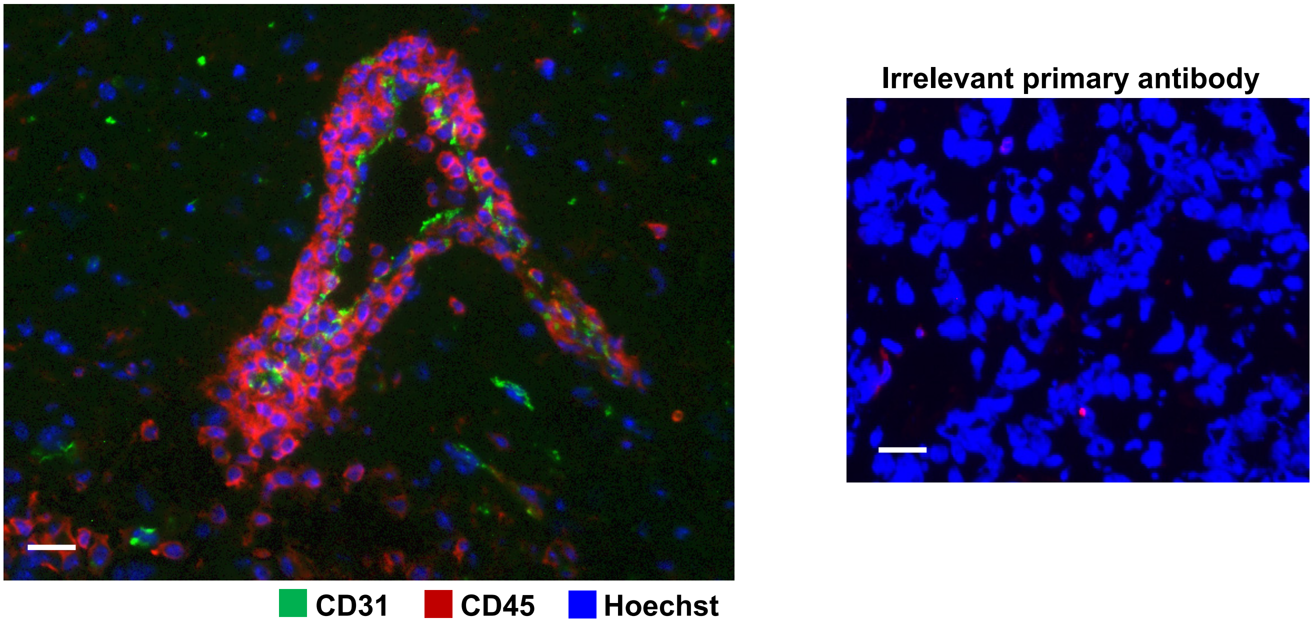

Figure S6. Perivascular leukocyte collections form around tumor blood vessels after treatment with standard-dose chemotherapy. GL261 tumors from WT host mice were harvested 5 days after a single dose of either: **A**, temozolomide (100 mg/kg, i.p.); or **B**, cyclophosphamide (100 mg/kg, i.p.). Tumors were frozen for immunohistochemical analysis of bone marrow-derived CD45-expressing leukocytes (red) and endothelial cells (CD31, green). Nuclei were counterstained with Hoechst (blue). Tumors stained with irrelevant primary antibody are included for reference. Representative photomicrographs of three tumors in each group are shown. Original magnification, x400; Scale bars, 25 μm.

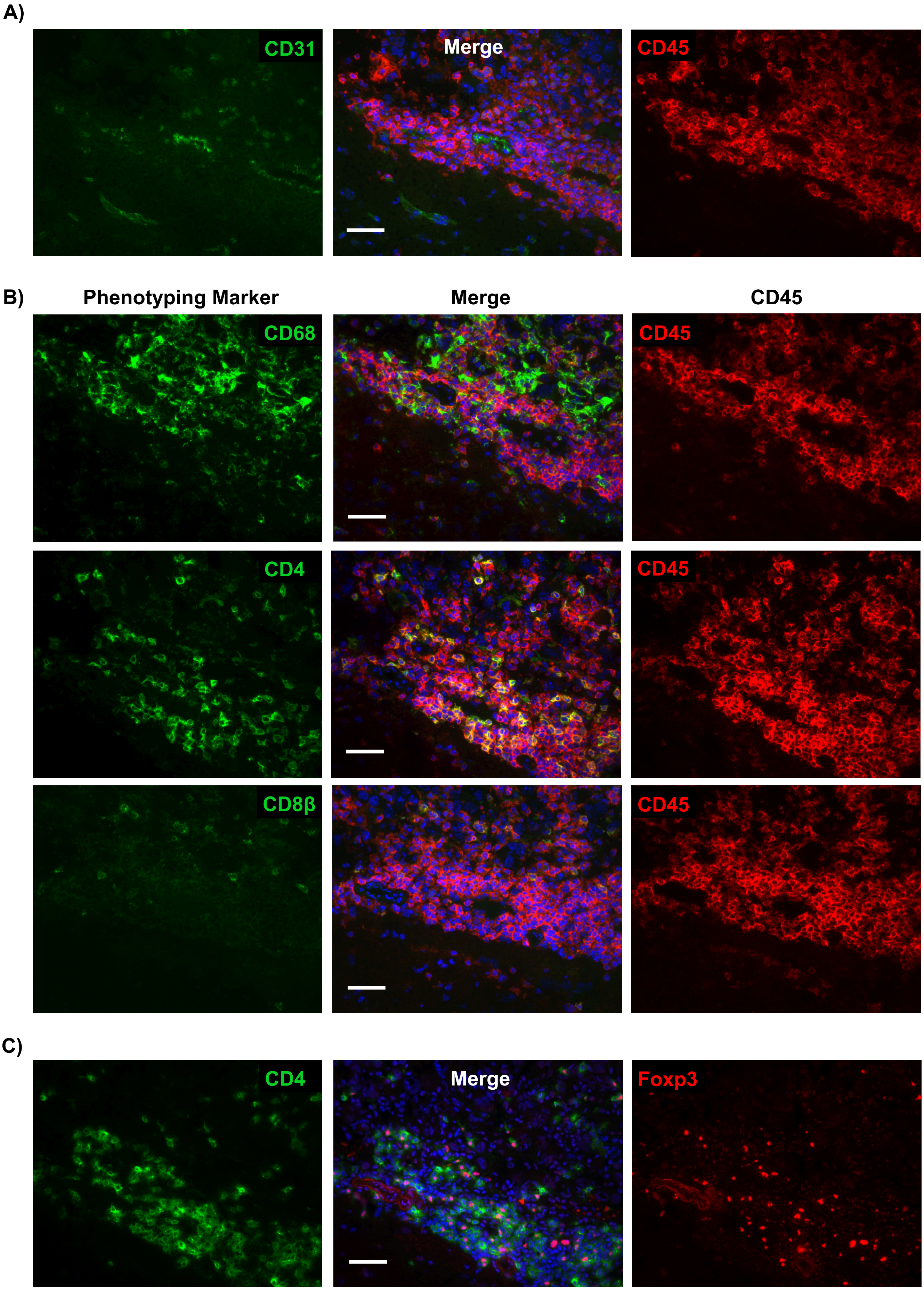

Figure S7. Macrophages, microglia and regulatory CD4 T cells predominate in perivascular leukocyte aggregates after chemotherapy.

Figure S7. Macrophages, microglia and regulatory CD4 T cells predominate in perivascular leukocyte aggregates after chemotherapy. GL261 tumors were harvested from WT host mice treated with low-dose temozolomide (25 mg/kg, i.p.). **A**, immunohistochemical analysis of bone marrow-derived CD45-expressing leukocytes (red) and endothelial cells (CD31, green) was used to identify large perivascular leukocyte cuffs. **B**, serial sections from these large cuffs were prepared and used for immunohistochemical phenotyping analysis of CD45-expressing leukocytes (red) co-stained with various phenotyping markers (in green) to identify macrophages and microglia (CD68, upper panels), CD4 T cells (CD4, middle panels), and CD8 T cells (CD8β, lower panels). **C**, a next-in-sequence tissue section was labeled with antibodies against CD4 (green) and Foxp3 (red). Nuclei were counterstained with Hoechst (blue). Photomicrographs are representative of cuff phenotyping studies of at least 3 tumors, from at least 3 independent experiments. Original magnification, x400; Scale bars, 25 μm.

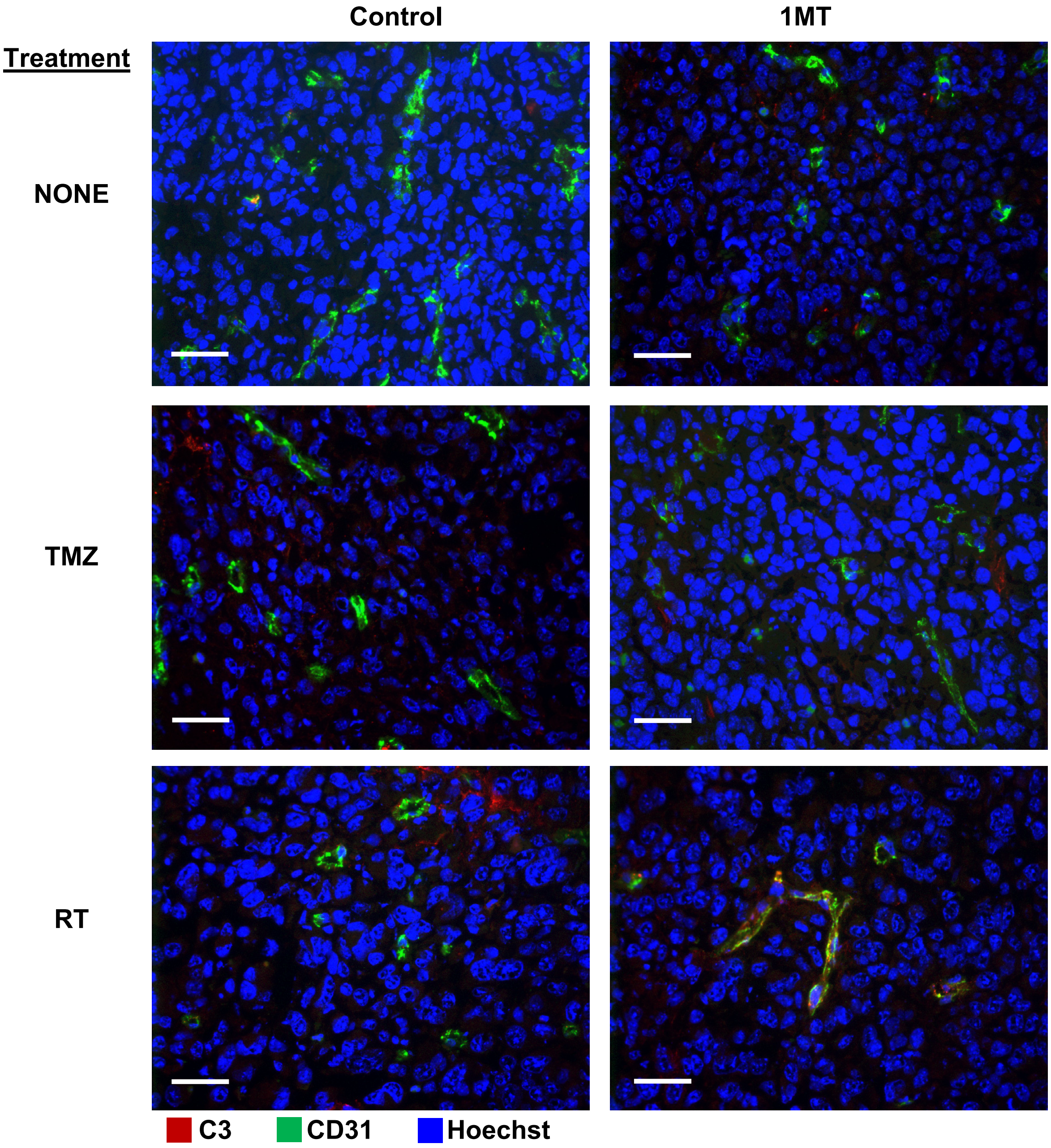

Figure S8. Complement deposition does not occur without the combination of IDO-pathway blockade, chemotherapy and radiotherapy. GL261 tumors were grown in syngeneic host mice treated with (right column) or without (left column) IDO-blockade using 1MT in drinking water continuously starting on day 14, and with either no additional treatment (upper panels), TMZ (100 mg/kg, i.p., middle panels) on day 16, or RT (500 cGy, lower panels) on day 17. Tumors were harvested on day 18 and frozen for immunohistochemical analysis of complement component C3 deposition (red) and endothelial cells (CD31, green). Nuclei were counterstained with Hoechst (blue). Data are representative of at least 3 mice per group, from at least 3 independent experiments. Original magnification, x400; Scale bars, 25 μm.

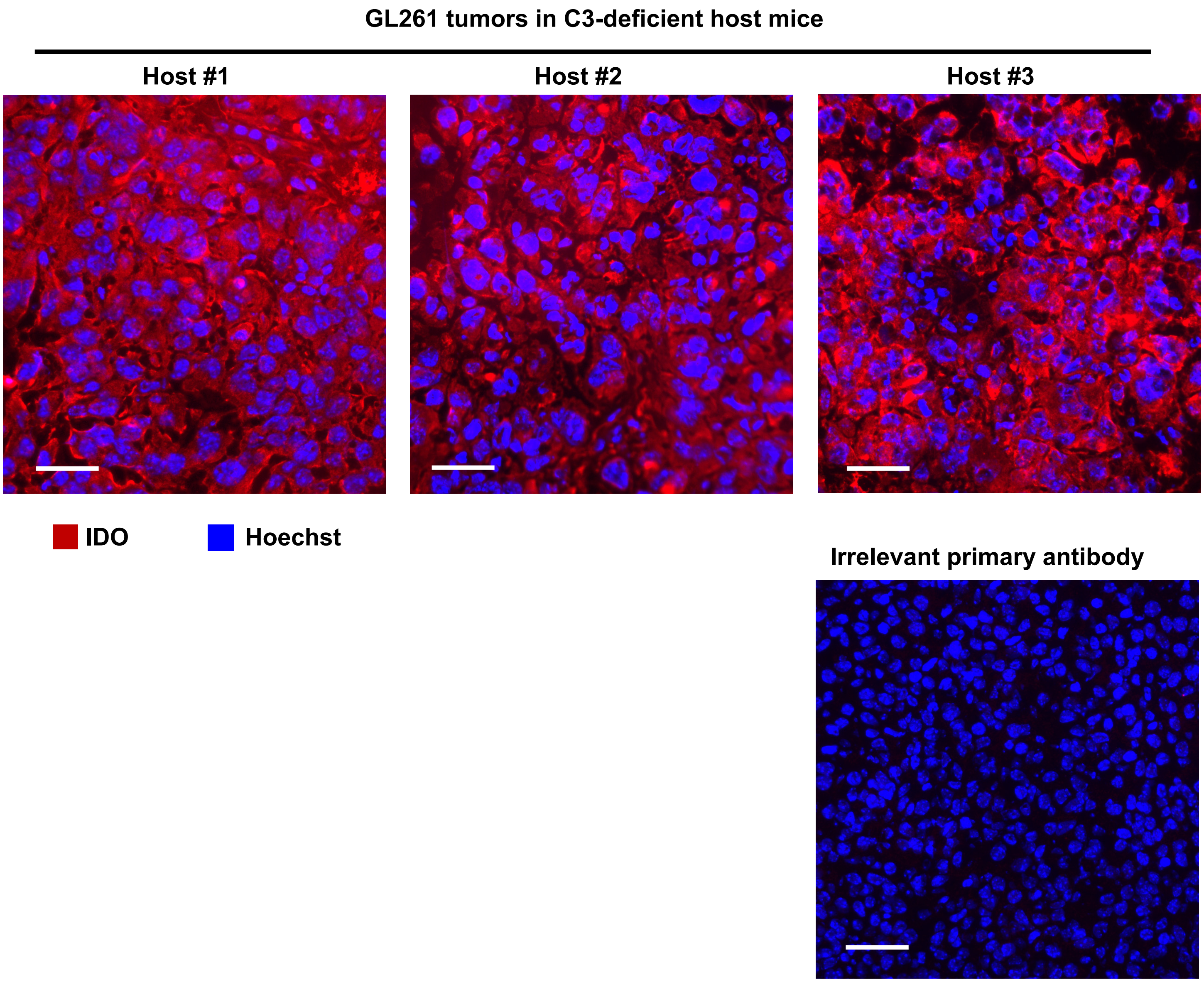

Figure S9. IDO is expressed by GL261 tumors grown in complement C3-deficient host mice. GL261 tumors were harvested from untreated syngeneic *C3*-/- host mice and frozen for immunohistochemical staining with polyclonal rabbit anti-mouse IDO (red) and nuclear counterstain (Hoechst, blue). Representative photomicrographs of three tumors are shown. Tumor stained with irrelevant primary antibody is included for reference. Original magnification x400; Scale bars, 50 μm.

Methods for Supplemental Figures

Cranial targeting of RT (500 cGy, Fig. S2) was performed using corporal shielding with the heads of anesthetized animals placed in the path of a 1 cm collimated radiation beam path. Immunohistochemical analyses were performed as follows. Primary antibodies for supplemental immunohistochemical studies were purchased from: SouthernBiotech [anti-mouse IDO polyclonal antibody raised in rabbit [1]]; Abcam [rabbit anti-mouse CD31 polyclonal antibody (catalog no. ab28364), rabbit anti-mouse Foxp3 polyclonal antibody (catalog no. ab54501)]; eBioscience [biotinylated rat anti-mouse CD45 (clone 30-F11), rat anti-mouse CD4 (clone RM4-5), rat anti-mouse CD8β (clone 53-6.7)], BioLegend [rat anti-mouse CD68 antibody (clone FA-11)]. Polyclonal rabbit anti-mouse IDO-2 antibody was a kind gift from Rick Metz at NewLink Genetics (Ames Iowa) [2]. Secondary immunohistochemistry reagents labeled with Alexa Fluor 488 or Cy3 were purchased from Jackson ImmunoResearch Laboratories, Inc. Tissue sections from frozen tumor blocks were fixed with ethanol and acetone (1:1), except for CD4 versus Foxp3 staining (methanol). For IDO stains (Figs. S4, S5, and S9), tissue was then incubated sequentially with unlabeled primary IDO-specific antibody, then with Cy3-labelled secondary antibody. For phenotyping of perivascular leukocyte cuffs (Fig. S7), fixed tissue was incubated sequentially with unlabeled primary antibodies, the corresponding secondary antibody, biotinylated anti-CD45, and Cy3-conjugated Streptavidin. Nuclear counterstain was performed using Hoechst (catalog no. 14530, Sigma-Aldrich).

References for Supplemental Methods

1. Sharma MD, Baban B, Chandler P, Hou DY, Singh N, Yagita H, Azuma M, Blazar BR, Mellor AL, Munn DH: **Plasmacytoid dendritic cells from mouse tumor-draining lymph nodes directly activate mature Tregs via indoleamine 2,3-dioxygenase.** *J Clin Invest* 2007, **117:**2570-2582.
2. Metz R, Duhadaway JB, Rust S, Munn DH, Muller AJ, Mautino M, Prendergast GC: **Zinc protoporphyrin IX stimulates tumor immunity by disrupting the immunosuppressive enzyme indoleamine 2,3-dioxygenase.** *Mol Cancer Ther* 2010, **9:**1864-1871.
